# Ultrasound study of right ventricular myocardial perfusion and functional changes in hypertrophic cardiomyopathy

**DOI:** 10.1186/s12872-024-03705-5

**Published:** 2024-01-22

**Authors:** Shan Cao, Lingjie Yang, Liyun Liu, Yuming Mu, Lina Guan

**Affiliations:** 1 Xinjiang Key Laboratory of Ultrasound Medicine, No. 137 Li Yu Shan South Road, Urumqi, China; 2 Department of Echocardiography, First Affiliated Hospital of Xinjiang Medical University, No. 137 Li Yu Shan South Road, Urmuqi, China

**Keywords:** Hypertrophic cardiomyopathy, Myocardial microcirculation, Myocardial contrast echocardiography, Speckle tracking

## Abstract

**Background:**

To evaluate the changes of right ventricular (RV) myocardial perfusion and function in patients with hypertrophic cardiomyopathy (HCM) by myocardial contrast echocardiography (MCE) and speckle tracking (2D-STE), and to explore the relationship between RV myocardial perfusion and strain.

**Methods:**

Conventional ultrasound, MCE and 2D-STE were performed on 29 HCM patients and 21 healthy subjects to analyze RV myocardial perfusion, RV global strain, RV free wall strain, and strain of each segment. The correlation between RV myocardial perfusion and strain was further analyzed in HCM patients.

**Results:**

MCE results showed that the regional myocardial perfusion of the RV in HCM patients was decreased. Compared with the normal control group, the mean slope (β) in the middle and apical segments of the RV free wall, and the peak intensity (A), β, myocardial blood flow (MBF) of the ventricular septum decreased in HCM patients (*P* < 0.05). RV function was impaired in HCM patients. The RV global strain (RV GLS), and the strain of RV free wall and each segment were lower than those in the normal control group (*P* < 0.05). Correlation analysis showed that there was a certain correlation between RV myocardial perfusion and strain, such as the β of the whole RV in HCM group had a positive correlation with the strain of the middle segment of the interventricular septum (*r* = 0.550, *P* = 0.002).

**Conclusions:**

The regional myocardial perfusion and strain of the RV in HCM patients are reduced, and there is a positive correlation between them, suggesting that the reduction of myocardial strain may be related to the impairment of myocardial microcirculation.

## Background

Hypertrophic cardiomyopathy (HCM) is a heart disease characterized [1, 2] by asymmetric hypertrophy of the ventricular muscle [3]. The prevalence of HCM in the population is high, with a worldwide incidence rate of about 1/500 and a mortality rate of 1%—2% [4, 5]. As the disease progresses, it often leads to damage to the structure and function of the heart. Although the early ejection fraction of HCM patients is preserved, local myocardial function is damaged due to changes in myocardial structure. The clinical prognosis of HCM is highly heterogeneous [6]. Heart failure, arrhythmia, and even sudden cardiac death may occur in the course of the disease, and its development is unpredictable. Studies have shown that HCM patients with normal coronary angiography still have different degrees of myocardial ischemia, which is essentially coronary microvascular dysfunction [7]. Coronary microcirculation disorders and the effect of reperfusion therapy and clinical prognosis [8, 9] can be accurately assessed by observing myocardial microcirculation perfusion [10] and assessing myocardial blood flow (MBF) with myocardial contrast echocardiography (MCE), which is of great importance to the clinical diagnosis and treatment of HCM.

At the same time, some studies have indicated that HCM may be a biventricular disease and that the left ventricle and right ventricle (RV) are functionally dependent on each other and affect each other. Previous studies on HCM were mostly confined to the left ventricle, and there were few studies on whether the RV of HCM was involved. Previous autopsy reports [11] have indicated that the RV wall in HCM patients showed significant thickening. The RV plays an important role in maintaining cardiac output, effective volume, and overall cardiac hemodynamics. Roşca M et al. [12] found that HCM patients with RV hypertrophy had an increased risk of sudden cardiac death. Thus, early detection and an understanding of HCM and the characteristics of RV myocardial microcirculation and functional changes can better determine individualized treatment plans for HCM patients.

In this study, two-dimensional speckle tracking echocardiography (2D STE) was used to measure the overall strain of the RV, the longitudinal strain of the RV free wall and the strain of each segment and to accurately assess the overall and local motion characteristics of the RV myocardium in patients with HCM. Our study was to evaluate the changes of right ventricular myocardial perfusion and function in HCM patients by using MCE and 2D-STE techniques, and to explore the relationship between right ventricular perfusion and strain.

## Methods

### Study participants

In this study, 29 patients diagnosed with HCM at the First Affiliated Hospital of Xinjiang Medical University from October 2019 to July 2021 were selected to be included in the HCM group. The diagnosis of HCM conformed to the clinical diagnostic criteria in the Guidelines for Clinical Application of Echocardiography in Diagnosis of Cardiomyopathy [13]: one or more segments of the left ventricle with a wall thickness of ≥ 15 mm and exclusion of secondary causes; first-degree relatives of patients with HCM; and detection of a single segment or multiple segments with a left ventricular wall thickness ≥ 13 mm during any cardiac imaging examination (echocardiography, CMR, CT, etc.) without other known causes. The exclusion criteria were as follows: patients with secondary ventricular wall hypertrophy caused by abnormal cardiac load; patients with obvious arrhythmia, poor image quality, incomplete clinical data, and anterior chest wall deformities; patients who did not meet the conditions for data analysis. In addition, 21 healthy subjects were matched to HCM patients during the same period and included as the control group. The normal controls had no evidence of hypertension and any other cardiovascular diseases. The results of the physical examination, electrocardiogram and echocardiography were all negative, and all control subjects were in sinus rhythm and without anterior chest wall deformities. This study was approved by the Ethics Committee of the First Affiliated Hospital of Xinjiang Medical University, and all subjects signed an informed consent form.

### Echocardiographic examinations

Imaging was performed using a Philips EPIQ7C color Doppler ultrasound diagnostic instrument and an S5-1 phased array probe with a frequency of 3.5 MHz and a frame rate of 55–90 frames/s. The subject was placed in the left lateral position, instructed to breathe calmly, and connected to the synchronous lead electrocardiogram. Then, routine echocardiography was performed, and at least 5 cardiac cycles were collected and recorded; these included the long-axis view of the left ventricle, the standard apical four-chamber view, the left parasternal RV inflow tract view, the subxiphoid view, and the apical four-chamber view dominated by the RV. Then, the imaging mode was switched to left ventricular output (LVO) (thermal index (TIS) = 0.7, mechanical index (MI) = 1.4) according to the imaging situation. SonoVue contrast agent (SonoVue, Bracco, Italy; a specification of SF6 gas, 59 mg, and lyophilized powder, 25 mg), which was diluted in normal saline to 5 ml and shaken well to form a milky white suspension, was injected intravenously. The above slices were observed and saved, and the dynamic images from the first 5 cardiac cycles during the "flash" mode to the 15 cardiac cycles after the flash were continuously recorded. With each flash, microbubbles are instantly broken at a high MI so that the intramyocardial contrast microbubbles are completely destroyed, and the refilling process of the myocardial microbubbles is observed. The above acquired images were saved on a CD-ROM for offline analysis using QLAB software.

### Image analysis

**Fig. 1:**
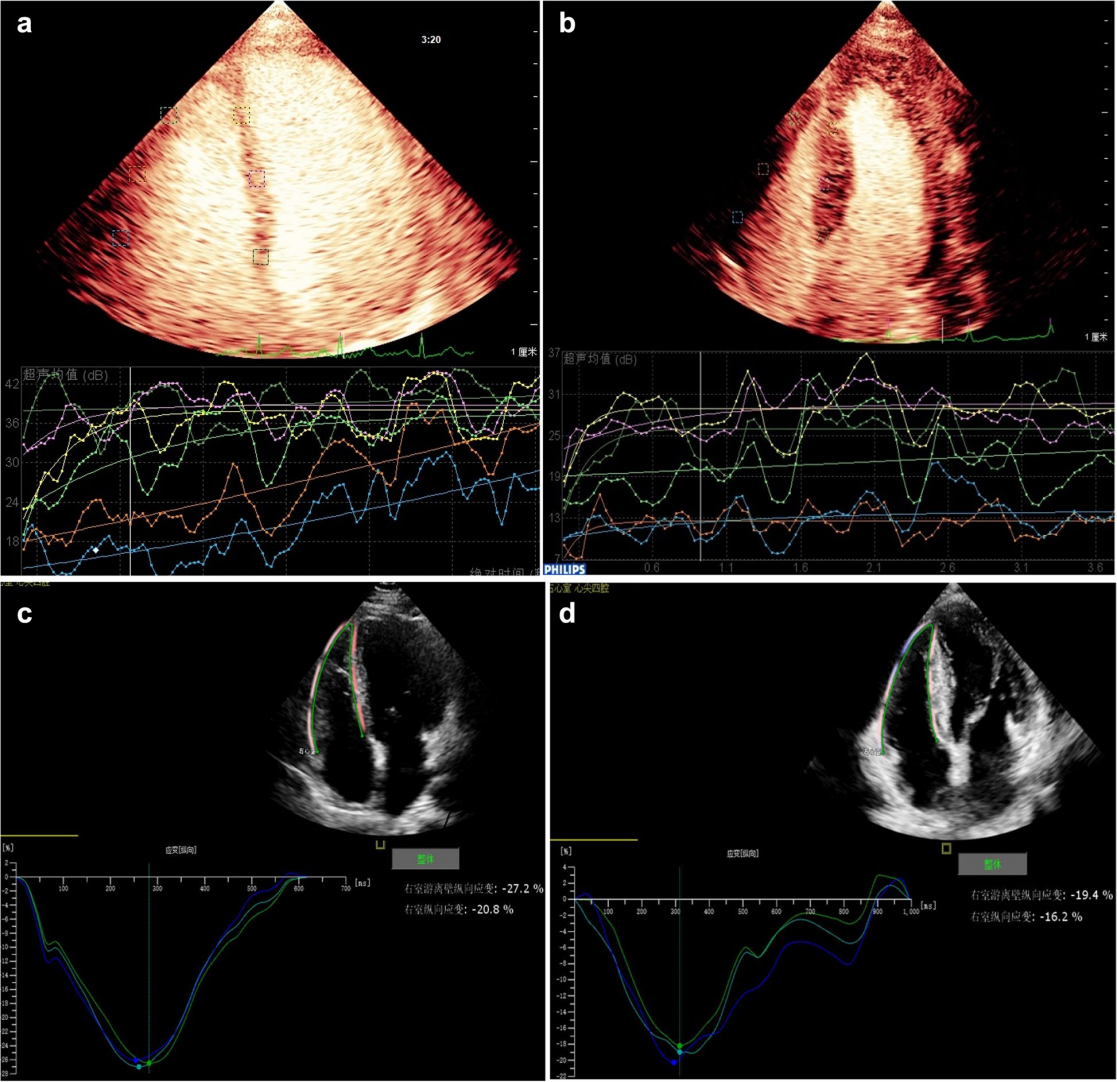
Typical right ventricular myocardial perfusion images of the normal subject (**A**) and the HCM patients (**B**) measured by MCE. Typical RV myocardial strain images of the normal subject (**C**) and the HCM patients (**D**) measured by 2D-STE

1. For conventional ultrasound data, routine echocardiography was used to obtain RV end-diastolic diameter, RV outflow tract diameter, RV wall thickness, interventricular septum thickness and left ventricular posterior wall thickness; the tricuspid regurgitation spectrum was used to estimate systolic pulmonary artery pressure (SPAP). The peak systolic velocity of the tricuspid valve annulus(S') was measured with tissue Doppler in the apical four-chamber view, and the Tei index was calculated from tissue Doppler sampling at the right ventricular free wall tricuspid annulus. Tricuspid annular longitudinal peak displacement (TAPSE) was measured with M-mode ultrasound, and the RV diastolic and end-systolic areas were measured with two-dimensional ultrasonography to calculate right ventricular fractional change (RV FAC).
2. For MCE, QLAB 10.8 software was used for analysis. The RV free wall and interventricular septum were divided into 6 segments, the region of interest (ROI) was placed in the ventricular wall of each segment near the endocardium; the correction tracking function was used to set the ROI position. Once the ROI was fixed, starting from the lowest acoustic intensity of the image after the flash, the ROI was dynamically tracked, the position of the sampling was adjusted frame by frame, and the changes in blood perfusion in the ROI were dynamically tracked. QLAB fits the curve according to the corresponding time of the myocardial acoustic signal intensity in the sampling frame, extracts the measured parameter value according to the fitting formula y = A (1- e—βt) that conforms to the contrast agent bolus injection mode—peak intensity (A) and the average slope of the curve (β)—and calculates the product of A × β. A, β and A × β represent myocardial blood volume, myocardial blood flow velocity and myocardial blood flow, respectively. (Fig. 1a, b)
3. For 2D-STE imaging analysis of RV systolic strain, the original image was imported into the QLAB workstation, the RV strain interface for quantitative cardiac motion analysis was accessed, and the RV was analyzed in the apical four-chamber view. The motion trajectory of the cardiac cycle and the motion curve were generated to obtain the right ventricular global longitudinal strain (RV GLS), the longitudinal strain of the RV free wall, and the strain values of each segment of the RV (Fig. 1c, d).

### Statistical analysis

All data were statistically analyzed by SPSS 23.0 software. Measurement data are expressed as the mean ± standard deviation $\left(\overline{x}\pm S\right)$, while count data are expressed as frequency or rate, according to the normality and homogeneity of variance. A t test was used for comparisons between two groups, and correlations were tested by Pearson’s correlation analysis. Data that did not conform to normality and homogeneity of variance were compared by the Mann–Whitney test, and correlations were tested by Spearman correlation analysis. The alpha level of all tests was *α* = 0.05; *P* < 0.05 was considered to be indicative of statistical significance.

### Intra- and interobserver variability

Reliability of myocardial perfusion and myocardial strain parameters were assessed by analyzing 10 randomly selected subjects. β, A, RV GLS and RV free wall strain were were measured by two experienced cardiologists who were blinded to patients′ clinical data results. Intraobserver variability was measured at different time. Interobserver variability was determined by repeating measurements from the same off-line images. Intra- and interobserver variability was calculated using intraclass correlation coefficients (ICCs).

## Results

### Clinical and echocardiographic characteristics

As shown in Table 1, there were 24 males and 5 females in the HCM group, with an average age of 47.28 ± 15.96 years, and 11 males and 10 females in the the control group, with an average age of 51.10 ± 11.45 years. There were no significant differences in age, height, weight, body surface area, systolic blood pressure, diastolic blood pressure or heart rate between the normal control group and the HCM group (*P* > 0.05).

**Table 1:** Clinical and Echocardiographic Characteristics $\left(\overline{x}\pm {\text{S}}\right)$

Variables		HCM (*n* = 29)	Control(*n* = 21)	*t*	*P*
Sex (n)	Male	24	11		
	Female	5	10		
Age (years)		47.28 ± 15.96	51.10 ± 11.45	-0.935	0.354
Height (cm)		171.59 ± 8.24	166.57 ± 9.53	1.989	0.052
Weight (kg)		74.90 ± 14.46	70.38 ± 12.30	1.159	0.252
Body surface area (m^2^)		1.87 ± 0.21	1.79 ± 0.20	1.333	0.189
Systolic blood pressure (mmHg)		121.69 ± 16.64	119.67 ± 13.72	0.456	0.651
Diastolic blood pressure (mmHg)		74.31 ± 9.89	74.29 ± 8.53	0.009	0.993
Heart rate		75.93 ± 6.18	72.62 ± 8.90	1.554	0.127
LVEF(%)		64.05 ± 2.39	63.61 ± 3.39	0.543	0.580
RVOT (mm)		28.24 ± 2.65	27.62 ± 2.56	0.831	0.410
RV end-diastolic diameter (mm)		18.72 ± 1.93	18.14 ± 1.80	1.083	0.284
RVWT (mm)		3.91 ± 0.25	2.72 ± 0.22	17.357	< 0.001
IVST (mm)		16.69 ± 4.99	9.19 ± 0.60	8.019	< 0.001
LVPWT (mm)		11.14 ± 1.38	8.90 ± 0.54	7.913	< 0.001
RV FAC (%)		49.89 ± 1.65	50.55 ± 0.75	-1.886	0.066
S' (cm/s)		13.92 ± 0.56	14.31 ± 0.85	-1.992	0.052
TAPSE(mm)		19.10 ± 1.20	23.04 ± 1.52	-11.206	< 0.001
RV Tei index		0.50 ± 0.05	0.35 ± 0.05	10.687	< 0.001
TR (m/s)		2.82 ± 0.28	2.56 ± 0.29	3.249	0.002

*Abbreviations*: *LVEF* left ventricular ejection fraction, *RVOT* right ventricular outflow tract, *RV* right ventricle, *RVWT* Right ventricular wall thickness, *IVST* interventricular septum thickness, *LVPWT* left ventricular posterior wall thickness, *RV FAC (%)* Right ventricular fractional change; S' (cm/s) Peak systolic velocity of tricuspid valve annulus, *TAPSE (mm)* Tricuspid annular plane systolic excursion, *TR (m/s)* Tricuspid regurgitation

Table 1 shows the comparison of conventional ultrasound parameters between two groups. Compared with those in the control group, the interventricular septum thickness, left ventricular posterior wall thickness, RV wall thickness, TR and RV Tei index were increased, and TAPSE was decreased in the HCM group (*P* < 0.001).

### Myocardial perfusion and strain of RV

As shown in Table 2, the average slope (β) of the middle and apical segments of the right ventricular free wall in the HCM group was lower than that in the normal control group (*P* < 0.05). Compared with those of the control group, the RV free wall, the overall mean slope (β) and MBF of the HCM group were all significantly decreased (*P* < 0.05).

**Table 2:** Right ventricular myocardial perfusion parameters

Variables			HCM (*n* = 29)	Control (*n* = 21)	*t/Z*	*p*
β/s-1	Whole RV		3.71 ± 1.39	6.25 ± 2.01	-5.275	< 0.001
	RV free wall		3.21 ± 1.46	4.81 ± 1.76	-3.503	0.001
	RV free wall	Base	3.28 ± 2.26	4.65 ± 3.19	-1.783	0.081
		Mid	3.14 ± 2.21	4.76 ± 3.10	-2.042	0.049
		Apical	3.21 ± 1.95	5.02 ± 3.18	-2.307	0.028
	Interventricular septum	Base	3.63 ± 2.54	7.50 ± 4.49	-3.561	0.001
		Mid	4.38 ± 2.43	7.43 ± 4.95	-2.599	0.015
		Apical	4.63 ± 3.27	8.12 ± 4.66	-3.117	0.003
A/dB	Whole RV		6.89 ± 1.90	8.52 ± 2.14	-2.838	0.007
	RV free wall		6.49 ± 2.02	7.49 ± 3.36	-1.212	0.235
	RV free wall	Base	6.26 ± 2.62	7.06 ± 3.73	-0.890	0.378
		Mid	6.42 ± 2.92	6.63 ± 3.79	-0.217	0.829
		Apical	6.77 ± 3.73	8.77 ± 5.65	-1.409	0.168
	Interventricular septum	Base	6.79 ± 3.97	8.85 ± 2.67	-2.065	0.044
		Mid	7.77 ± 3.72	9.78 ± 3.01	-2.039	0.047
		Apical	7.32 ± 3.07	10.05 ± 3.23	-3.037	0.004
MBF/(dB·s-1)	whole RV		29.13 ± 15.35	53.72 ± 23.87	-3.921	< 0.001*
	RV free wall		22.44 ± 13.41	32.24 ± 17.12	-1.975	0.048*
	RV free wall	Base	19.97 ± 17.67	27.46 ± 17.12	-1.897	0.058*
		Mid	21.55 ± 17.18	28.74 ± 22.84	-0.934	0.350*
		Apical	25.81 ± 25.25	40.53 ± 36.28	-1.700	0.089*
	Interventricular septum	Base	29.06 ± 29.63	73.23 ± 60.47	-3.253	0.001*
		Mid	39.17 ± 34.94	73.12 ± 59.60	-2.565	0.010*
		Apical	39.19 ± 35.04	79.27 ± 42.11	-3.273	0.001*

*Abbreviations*: *RV* right ventricle, *A/dB* peak intensity, *β/s-1* average slope of the curve, *MBF/(dB·s-1)* myocardial blood flow

^*^Mann–Whitney test

Table 3 shows the comparison of RV strain parameters between the HCM group and the control group. RV GLS in HCM group (-16.66 ± 2.31%) decreased compared with the control group (- 21.25 ± 2.35%), and the longitudinal strain of the free wall (-18.35 ± 2.88%) was also lower than that of the control group (-21.78 ± 4.17%) (*P* < 0.05). The strains of each segment (basal, middle and apical segment) of the RV free wall and the ventricular septum in the HCM group decreased compared with the control group (*P* < 0.05).

**Table 3:** Right ventricular strain parameters

Observation Indicator	HCM (*n* = 29)	Control(*n* = 21)	*t*	*P*
RV GLS	-16.66 ± 2.31	-21.25 ± 2.35	6.869	< 0.001
RV free wall strain	-18.35 ± 2.88	-21.78 ± 4.17	3.254	0.003
Free wall basal segment strain	-19.47 ± 3.25	-23.01 ± 4.77	2.945	0.006
Free wall midsection strain	-18.17 ± 2.94	-21.17 ± 4.69	2.582	0.015
Free wall tip strain	-17.41 ± 3.32	-21.17 ± 4.35	3.467	0.001
Basal segment strain of the interventricular septum	-14.70 ± 2.30	-19.01 ± 2.38	6.449	< 0.001
Mid-septal strain	-14.52 ± 3.30	-21.38 ± 2.05	9.050	< 0.001
Strain in the apical segment of the interventricular septum	-15.71 ± 3.31	-21.77 ± 2.27	7.237	< 0.001

*Abbreviations*: *RV GLS (%)* Right ventricular global longitudinal strain

### Correlation analysis of myocardial perfusion and strain of RV

Correlation analysis showed that the overall peak intensity (A) of the RV was positively correlated with the strain of the apical segment of the RV free wall (*r* = 0.484, *P* = 0.008), and the mid-septal peak intensity was positively correlated with RV GLS (*r* = 0.381, *P* = 0.042) (Table 4). The overall β of the RV was positively correlated with the strain of the middle ventricular septum (*r* = 0.550, *P* = 0.002) (Table 5). The MBF of the basal segment of the interventricular septum were positively correlated with the strain of the basal segment of the interventricular septum (*r* = 0.404, *P* = 0.030) (Table 6).

**Table 4:** Correlation analysis between peak strength (A) and strain parameters

Perfusion A (dB)			Whole right ventricle (A)	Right ventricular free wall (A)	Right ventricular free wall segment (A)			Interventricular segment (A)		
Strain (%)					Base	Mid	Apical	Base	Mid	Apical
RV GLS		*r*	0.249	-0.038	-0.040	-0.010	-0.026	0.312	0.381^▲^	0.135
Right ventricular free wall		*r*	0.269	0.024	0.064	0.035	-0.033	0.323	0.218	0.069
Right ventricular free wall	Base	*r*	0.044	-0.061	-0.029					
	Mid	*r*	0.197	-0.065		-0.056				
	Apical	*r*	0.484^▲^	0.182			0.078			
Interventricular septum	Base	*r*	0.334	-0.043				0.343		
	Mid	*r*	0.095	-0.194					0.299	
	Apical	*r*	0.014	0.002						-0.090

^▲^ Statistical significance (P < 0.05) is indicated by triangles

**Table 5:** Correlation analysis of the average slope (β) of the curve and strain parameters

Perfusion β(s-1)			Whole right ventricle (β)	Right ventricular free wall (β)	Right ventricular free wall segment (β)			Interventricular segment (β)		
Strain (%)					*Base*	*Mid*	*Apical*	*Base*	*Mid*	*Apical*
RV GLS		*r*	-0.012	0.002	0.003	0.117	-0.132	0.017	0.089	-0.112
Right ventricular free wall		*r*	-0.215	-0.107	-0.055	0.035	-0.181	-0.036	0.081	-0.251
Right ventricular free wall	Base	*r*	-0.197	-0.079	0.016					
	Mid	*r*	-0.152	-0.080		0.119				
	Apical	*r*	-0.043	-0.091			-0.116			
Interventricular septum	Base	*r*	0.334	0.130				0.235		
	Mid	*r*	0.550^*^	0.133					0.112	
	Apical	*r*	0.330	0.125						-0.084

^*^Indicating *P < *0.05

**Table 6:** Correlation analysis between myocardial blood flow (MBF) and strain parameters

Perfusion MBF/(dB·s-1)			Whole right ventricle (MBF)	Right ventricular free wall (MBF)		ght ventricular free wall segment (MBF)			Interventricular segment (MBF)		
strain (%)						Base	Mid	Apical	Base	Mid	Apical
RV GLS		r	0.079		0.053	-0.020	0.206	-0.040	0.188	0.159	-0.014
Right ventricular free wall		r	-0.068		-0.005	0.002	0.174	-0.071	0.103	0.073	-0.185
Right ventricular free wall	Base	r	-0.169		-0.032	0.076					
	Mid	r	-0.090		-0.030		0.170				
	Apical	r	0.214		0.110			0.048			
Interventricular septum	Base	r	0.379^*^		0.136				0.404^*^		
	Mid	r	0.192		-0.064					0.178	
	Apical	r	-0.136		0.104						-0.129

^*^Indicating *P* < 0.05

### Reproducibility detection

The results for the intraobserver and interobserver variability for the myocardial perfusion and myocardial strain parameters upon repeated measurements in 10 random patients were shown in Table 7. ICC values were high for intra-observer and interobserver variability when the same images were analyzed by the two different cardiologists. Besides, the results also demonstrated that the analysis of the software had the well repeatability and reliability in this study (Table 7).

**Table 7:** Analysis of intra- and inter-observer agreement of myocardial perfusion and myocardial strain parameters

Variables	Intra-observer			Intero-bserver		
	ICC	95% CI	*p*	ICC	95% CI	*p*
β/s-1	0.898	0.614–0.974	0.001	0.864	0.385–0.967	0.001
A/dB	0.916	0.655–0.979	< 0.001	0.892	0.413–0.975	< 0.001
MBF/(dB·s-1)	0.883	0.365–0.973	< 0.001	0.777	-0.207–0.952	0.001
RV GLS	0.965	0.444–0.993	< 0.001	0.864	0.387–0.967	0.001
RV free wall strain	0.926	0.187–0.986	< 0.001	0.829	0.143–0.960	0.001

## Discussion

HCM is the most common inherited cardiomyopathy worldwide with a prevalence of 0.16% to 0.29% in the general population [14]. Although the ESC definition of HCM is based on LV wall thickness criteria (15 mm in one or more myocardial segments that is not explained solely by loading conditions), RV involvement can be noteworthy, as it is not rare and can affect the prognosis of the disease [14]. GLS is the optimal tool for assessment of subclinical ventricular dysfunction. Some studies have found a decrease in RV GLS in HCM patients [12], however, data evaluating RV GLS and myocardial perfusion simultaneously in HCM patients is limited, and the relationship between the two is unclear. In our study, MCE and 2D-STE were used to focus on the changes of right ventricular myocardial perfusion and strain in HCM patients. The study found that the RV function of HCM patients was impaired. The local myocardial perfusion and the strain of the RV were reduced, and there was a correlation between them.

The RV free wall and septum are the main components of the RV. The results of MCE showed that the interventricular septal myocardial perfusion decreased in HCM patients, and the β values of the RV free wall midsection and apical segment were also reduced. The dysfunction of myocardial microcirculation in HCM patients may be due to hypertrophy of myocardial cells, proliferation of myocardial interstitium and fibrous tissue, which leads to the decrease of myocardial capillary density, increase of vascular resistance and decrease of myocardial blood volume. Some scholars found that the middle membrane of small arteries in the ventricular wall of HCM patients was significantly thickened, and the lumen was narrowed or even occluded through autopsy [15], which change may be the pathological basis for the decrease of coronary microvessel density in HCM myocardium. Myocardial perfusion may decrease with the increase of ventricular wall thickness. In our study, the right ventricular wall of HCM patients is thickened, and the myocardial perfusion of the RV free wall is decreased, which may be because the RV free wall myocardium is also infiltrated by the fibrous tissue of the adjacent ventricular septum [16, 17].

2D-STE can be used to calculate the deformation degree of each segment of myocardium in the region of interest by tracking the motion track of myocardial tissue. The clinical advantage of strain remains in its higher sensitivity to subtle functional changes. Sophie et al. assessed the usefulness of a longitudinal strain of LV adjusted to regional thickness in HCM, and proved that longitudinal strain adjusted to regional thickness of LV give us information about fibrosis’ burden as in segments affected by significant myocardial fibrosis it is lower than conventional strain [18]. Nestor et al. found that left atrial strain and strain rate can be used to identify diastolic dysfunction and low left atrial reservoir and conduit strain are associated with adverse outcomes in HCM patients [19]. To date, RV 2D-STE has been used as an objective and accurate tool in the evaluation of RV function, and is a valuable asset in recognizing early subclinical changes in the myocardium. Even if 3D-strain imaging echocardiography and cardiac magnetic resonance-feature tracking (CMR-FT) are more sensitive diagnostic tools whose extensive use is needed [20, 21]. Because 80% of RV ejection is mainly completed by longitudinal myocardial fiber contraction, RV systolic function is reflected by the longitudinal strain of the RV [11]. D'Andrea et al. [22] found that the RV strain in HCM patients was reduced and that it was closely related to impaired RV systolic function, suggesting that the decrease of RV strain may may indicate early subclinical myocardial injury. The results showed that the overall strain of the RV, and the local strain of the ventricular septum and free wall in HCM patients were lower than those of normal people, indicating that RV systolic function was impaired in HCM patients. It is worth noting that some factors may influence myocardial strain, such as diabetes, dialysis, obesity and some drug therapy (such as beta-blockers) through negative inotropic and chronotropic effects [23]. Besides, 2D-STI results may be hampered by the possible influence of anterior chest wall deformities on cardiac kinetics [24]. In our study, there was no statistically significant difference in BMI between the two groups, which can exclude the influence of obesity on myocardial strain. However, more clinical factors need to be considered, such as diabetes, dialysis, and drugs taken that may affect myocardial strain. Conventional ultrasound detection revealed that TAPSE decreased and RV Tei index increased, indicating that both the systolic and diastolic functions of the RV in patients with HCM were damaged, which was basically consistent with the results of previous studies.

The results of correlation analysis showed that the basal and mid-segment strains of the interventricular septum were correlated with RV perfusion, and the strains of the apical segment of the RV free wall was correlate with RV global peak intensity (A), indicating that the mechanical changes of the right ventricle in HCM were related to the right ventricular perfusion. Wang et al. [25] found that the reduction in the myocardial systolic function of HCM patients is related to regional perfusion injury. The myocardial ischemia caused by regional myocardial perfusion injury in HCM patients may cause myocardial fibrosis and changes in the myocardial structure, leading to reduced myocardial deformation ability and impaired myocardial strain. Our findings may apply to the field of cardiac oncology for early detection of right ventricular dysfunction and cardiac toxicity caused by anti-tumor drugs [26].

Limitations: There are also some limitations in the study. Firstly, 2D-STI requires high-quality 2D images and high frame rate to ensure the accuracy of its measurements, which increases the difficulty of the examination. And 2D-STI cannot reflect the stereoscopic movement of the heart, which may result in spot loss and affect the accuracy of the measurement [27]. Furthermore, myocardial perfusion parameters are are also easily affected by various factors, such as image quality and intravenous injection microbubble velocity. Although we carried out strict quality control during image acquisition and data analysis, some errors may remain. Secondly, the sample size of this study was small and the number of female patients in the HCM group was relatively small. We will expand the sample size to balance the gender of patients in the study and further explore the changes in RV myocardial perfusion and function in patients with different HCM classifications. In addition, the duration of this study was short, and there were no follow-up or prognostic parameters. Thus, supplementation with follow-up data in the future is necessary to further study the influence of right ventricular perfusion and functional changes on the prognosis of HCM patients.

## Conclusion

In conclusion, our study focused on the changes of RV in HCM patients, and analyzed the changes of RV myocardial perfusion, as well as the characteristics of global or local myocardial motion using MCE and 2D-STE technology. The study confirmed that the RV function of HCM patients was impaired. The local myocardial perfusion and the strain of the RV were reduced, and there was a correlation between them, suggesting that the reduction of myocardial strain may be related to the damage of myocardial microcirculation, which is of great significance for clinical understanding of HCM cardiac injury.

## Data Availability

The datasets used and/or analysed during the current study available from the corresponding author on reasonable request.

## Abbreviations

RV: Right ventricular
HCM: Hypertrophic cardiomyopathy
MCE: Myocardial contrast echocardiography
2D-STE: Speckle tracking
MBF: Myocardial blood flow
RV GLS: RV global strain
LVO: Left ventricular output
TIS: Thermal index
MI: Mechanical index
SPAP: Systolic pulmonary artery pressure
TAPSE: Tricuspid annular longitudinal peak displacement
RV FAC: Right ventricular fractional change
ROI: Region of interest
A: Peak intensity
β: The average slope of the curve
LVEF: left ventricular ejection fraction
